# Complicated Sinus of Valsalva Aneurysm Initially Diagnosed as Atrial Myxoma

**DOI:** 10.5935/abc.20160126

**Published:** 2016-09

**Authors:** Rafael Plens Teixeira, Pedro Felipe Gomes Nicz, Felipe Lourenço Fernandes, Renner Augusto Raposo Pereira, Roney Orismar Sampaio, Flavio Tarasoutchi

**Affiliations:** Instituto do Coração, Hospital das Clínicas, Faculdade de Medicina, Universidade de São Paulo - USP, São Paulo, SP, Brazil

**Keywords:** Aortic Aneurysm / surgery, Sinus of Valsalva, Aortic Valve Insufficiency / surgery, Mitral Valve Insufficiency / Surgery, Echocardiography, Transesophageal, Magnetic Resonance Spectroscopy

## Introduction

Atrial myxoma and sinus of Valsalva aneurysm (SVA) are rare conditions and possibly underdiagnosed in clinical practice. We report an unusual presentation of a left SVA after an episode of infective endocarditis (IE). The SVA extrinsically compressed the left main coronary artery (LMCA), which was initially diagnosed as a left atrial myxoma.

## Case Report

**Figure 1:**
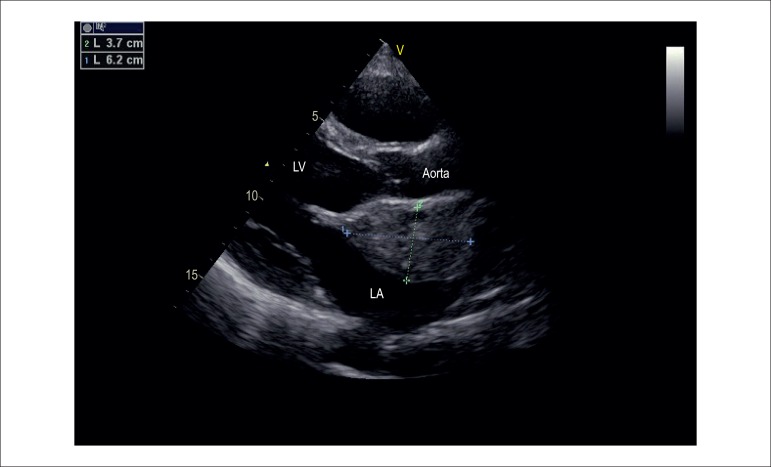
Transthoracic Echocardiography with image suggestive of left atrial myxoma. LV: left ventricle; LA: left atrium.

Male patient, 51 years old, was referred to our clinic for evaluation of aortic and mitral valve dysfunction after an IE by *Streptococcus viridans,* which was medically treated. The patient did not have any personal history and his only symptom was dyspnea on exertion. Physical examination and transthoracic echocardiogram (TTE) were consistent with significant aortic and mitral regurgitation, and the TTE disclosed an image suggestive of a left atrial myxoma measuring 6.2 x 3.7 cm (Figure 1) and a posterior eccentric mitral regurgitation jet.

Therefore, surgical procedure for aortic and mitral valve replacement was indicated, as well as for removal of the myxoma. Preoperative tests were performed, including cardiac catheterization, transesophageal echocardiography (TEE) and cardiac magnetic resonance (CMR).

**Figure 2:**
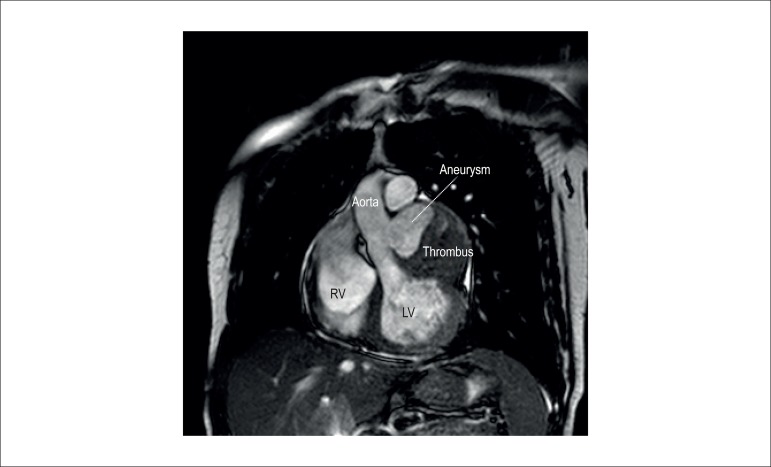
Cardiac Magnetic Resonance showing the presence of sinus of Valsalva aneurysm filled with thrombus. RV: right ventricle; LV: left ventricle.

The catheterization showed important extrinsic compression of the LMCA by a left SVA that had gone undetected. Both the TEE and the CMR (Figure 2) suggested that this aneurysm was filled with thrombus. Biventricular dysfunction, moderate thickening of the aortic and mitral valves and presence of a hyperechoic image in the anterior mitral leaflet were also observed.

Therefore, the hypothesis of atrial myxoma was ruled out.

The surgery consisted in a safety graft of a saphenous vein graft bypass to the anterior descending artery; the removal of 180 g of aneurysm thrombus; and its occlusion using a bovine pericardium patch. Moreover, 50 g of thrombus adjacent to the anterior mitral leaflet were also removed, where a sinus of Valsalva fistula was found and closed. Finally, mitral and aortic valve replacement was performed, using bioprostheses. According to the subsequently performed histological examination, the previous findings were all suggestive of IE sequel.

## Discussion

SVA are usually congenital and more frequent in the right sinus (65 to 85%), followed by non-coronary ones (10 to 30%) and those located in the left sinus (< 5%).^1^ According to some researchers, left sinus aneurysms are most frequently acquired and can be caused by atherosclerosis or be the sequelae of diseases such as syphilis or IE.^2^ These aneurysms can rupture and become life-threatening. The rupture usually occurs into the heart chamber adjacent to the affected sinus. Its most common complications are aortic regurgitation and ventricular septal defect.^3,4^ LMCA stenosis can also occur due to extrinsic compression. In this case, both LMCA compression and rupture (fistula) occurred.

Because of the close association between the thrombus and the left atrium, the SVA was initially diagnosed as a myxoma.

The urgent surgical repair is recommended in patients with SVA rupture, especially in the event of intracardiac shunts.^5,6^ For the other types of SVA, surgical repair is generally recommended due to progressive association with poor prognosis. Surgery has satisfactory results and low morbidity and mortality related to the procedure (2%).^7,8^

In our case, the delay in attaining the correct diagnosis may have exposed the patient to a greater risk for complications.

## Conclusion

Transesophageal Echocardiography should not be used alone for the detection of post-endocarditis complications. Unusual complications might go undetected. Transesophageal Echocardiography is mandatory and Cardiac Magnetic Resonance may be of great usefullness.^4,9^
